# Laser light triggers increased Raman amplification in the regime of nonlinear Landau damping

**DOI:** 10.1038/ncomms5158

**Published:** 2014-06-18

**Authors:** S. Depierreux, V. Yahia, C. Goyon, G. Loisel, P. -E. Masson-Laborde, N. Borisenko, A. Orekhov, O. Rosmej, T. Rienecker, C. Labaune

**Affiliations:** 1 CEA, DAM, DIF, F- 91297 Arpajon, France; 2 LULI, UMR 7605 CNRS-Ecole Polytechnique-CEA-Université Paris VI, 91128 Palaiseau Cedex, France; 3 P. N. Lebedev Physical Institute, 53 Leninskii Prospect, Moscow 119991, Russia; 4 Helmotzzentrum fur Schwerionenforschung GSI, 64291 Darmstadt, Germany

## Abstract

Stimulated Raman backscattering (SRS) has many unwanted effects in megajoule-scale inertially confined fusion (ICF) plasmas. Moreover, attempts to harness SRS to amplify short laser pulses through backward Raman amplification have achieved limited success. In high-temperature fusion plasmas, SRS usually occurs in a kinetic regime where the nonlinear response of the Langmuir wave to the laser drive and its host of complicating factors make it difficult to predict the degree of amplification that can be achieved under given experimental conditions. Here we present experimental evidence of reduced Landau damping with increasing Langmuir wave amplitude and determine its effects on Raman amplification. The threshold for trapping effects to influence the amplification is shown to be very low. Above threshold, the complex SRS dynamics results in increased amplification factors, which partly explains previous ICF experiments. These insights could aid the development of more efficient backward Raman amplification schemes in this regime.

## Abstract (web summary)

Stimulated Raman scattering is one of the methods being explored to generate ultrahigh intensity short laser pulses. Depierreux *et al*. explore a new regime, also relevant to inertial confinement thermonuclear fusion, in which nonlinear kinetic response of a hot plasma enhances Raman amplification.

## Graphical abstract

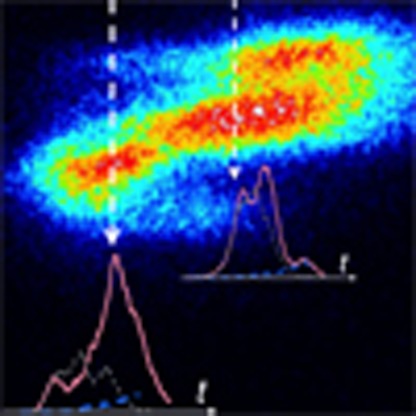

Resonantly excited plasma normal modes have long provided a means to study space plasmas^1^,^2^,^3^. For example, measuring the characteristic frequency of Langmuir waves gives access to the electron density^4^. The development of high-intensity lasers has opened new areas of applications for the excitation of Langmuir waves in plasma, now widely used to produce high-amplitude accelerating electric field^5^,^6^,^7^ or to transfer energy between laser electromagnetic waves^8^,^9^,^10^,^11^. The plasma medium is used here because it can sustain very large electric fields without damage. Although the potential to excite large amplitude electron plasma waves (EPWs) with lasers is evident, the description of the plasma response is less obvious and must include nonlinear effects. Additional complications come from currently higher available plasma temperature, which results in a significant amount of electrons approaching the phase velocity of the Langmuir wave. Then, the fluid approach no longer applies and the EPW will experience collisionless Landau damping^12^ for which an exact mathematical treatment exists only for infinitesimal amplitude plasma waves^13^. As the EPW amplitude increases, trapping of the electrons in its potential well significantly modifies the electron distribution function in the neighbourhood of the EPW phase velocity, causing a reduction of the Landau damping rate compared with its linear value. This reduction has been measured in low-temperature plasmas (<10 eV)^14^,^15^.

Because of its inherent complexity, nonlinear Landau damping is still the subject of theoretical and numerical studies^16^,^17^,^18^,^19^,^20^ more than 50 years after the Landau’s initial calculation. For laser-driven Langmuir waves, the consequences of trapping are even more complicated because additional opposite effects can simultaneously occur. The trapping-induced modification of the distribution function also produces a nonlinear shift of the frequency^21^,^22^ of the EPW that can detune the resonance and finally reduce the coupling of the EPW with its laser drive. Additional two- and three-dimensional trapped particle instabilities may also limit the EPW amplitude in this case^23^,^24^. These nonlinear trapping effects affecting a driven EPW in high-temperature plasmas have been studied theoretically and numerically^25^,^26^,^27^,^28^,^29^,^30^,^31^,^32^,^33^,^34^, but some critical trapping features remain difficult to track with simulations representative of experimental conditions.

Here we present direct measurements of the nonlinear behaviour of driven Langmuir waves in high-temperature plasmas and of its impact on the Raman amplification factor. The experiment uses laser beams in a three steps procedure: (i) a broadband electromagnetic wave is first generated by the resonant interaction of the incident laser with a target, (ii) an EPW is produced by optical mixing of this counterpropagating wave with the incident laser in a preformed plasma and (iii) the evolution of the driven EPW is deduced from the Raman amplification factor of this plasma. Trapping nonlinearities are evidenced in the driven EPW response by comparing the stimulated Raman scattering (SRS) of the plasma with and without the seed. The threshold intensity of the counterpropagating seed beam above which trapping nonlinearities develop is directly measured in the experiment and found to be lower than 10^12^ W cm^−2^. Above threshold, we demonstrate a sharp increase in the Raman amplification factor at scattered light wavelengths for which linear Landau damping was dominant. This allows us to identify a laser and plasma parameter region for which kinetic nonlinearities in the Langmuir waves result in increased Raman amplification. The plasma parameters are relevant to previously reported backward Raman amplifier (BRA)^35^,^36^ and inertial confinement fusion (ICF) experiments^37^,^38^,^39^. The level of seed intensity that triggers nonlinearities in the plasma wave response corresponds to low levels of SRS reflectivities inside ICF targets and is close to the seed intensity available in BRA experiments.

## Results

### Experimental setup

**Figure 1:**
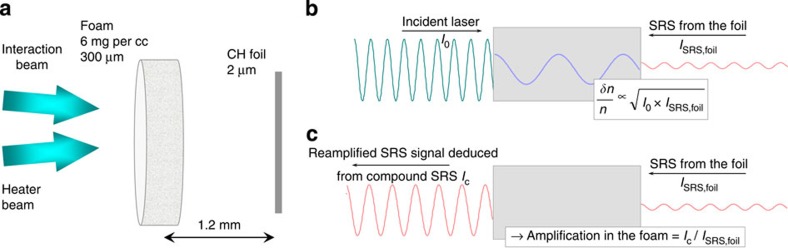
The compound target configuration. (**a**) Sketch of the experimental setup: the heating beam first ionizes and heats the foam before the interaction beam starts; (**b**) the incident interaction laser beam (of intensity *I*_0_ and frequency *ω*_0_) and the SRS light backscattered by the foil (of intensity *I*_SRS,foil_ and frequency *ω*_SRS_) couple via the ponderomotive force to drive an EPW in the foam plasma (of frequency *ω*_EPW_=*ω*_0_−*ω*_SRS_) whose amplitude is controlled by the product *I*_0_ × *I*_SRS,foil_; (**c**) the SRS from the foil is amplified as it propagates through the foam allowing the measurement of the amplification factor of the foam.

The seeded experiment uses a compound target (see Fig. 1a) made up of an underdense foam followed by a 2-μm-thick CH foil. In the compound target, the CH foil generates a backscattered SRS signal propagating backward in the foam plasma, where it beats with the incident laser electromagnetic wave to drive an EPW. This initial situation is shown in Fig. 1b. Backscattering measured from the compound target provides information about the amplification of the foil SRS signal in the foam plasma (see Fig. 1c). The foam and foil responses are also studied individually for comparison: (i) the seed signal is measured with shots performed with the foil alone and (ii) shots with the foam alone give the SRS contribution of the foam when starting from thermal noise. The latter provides the intrinsic amplification of the foam plasma resulting from its hydrodynamic expansion as a function of time.

The experiment uses two consecutive beams of 1.5 ns duration and 400 J energy at 526.5 nm. The first beam is used to ionize and heat the low-density TAC (C_12_H_16_O_8_) foam^40^, producing a fully ionized plasma by the end of the heater pulse^41^. The foams used in this experiment have a 6 or 3 mg cc^−1^ density and are 300 μm long. At *t*=1.5 ns, they are heated up to (0.8±0.2) keV electron temperature as deduced from the intensity ratio of H-like and He-like lines observed in the spectrally resolved X-ray emission of a chlorine dopant added at the 1% level for diagnostic purposes. The interaction is studied on the second beam. Experimental details on the beams and diagnostics configuration are given in the Methods section.

### Experimental results in the foam-alone target

**Figure 2:**
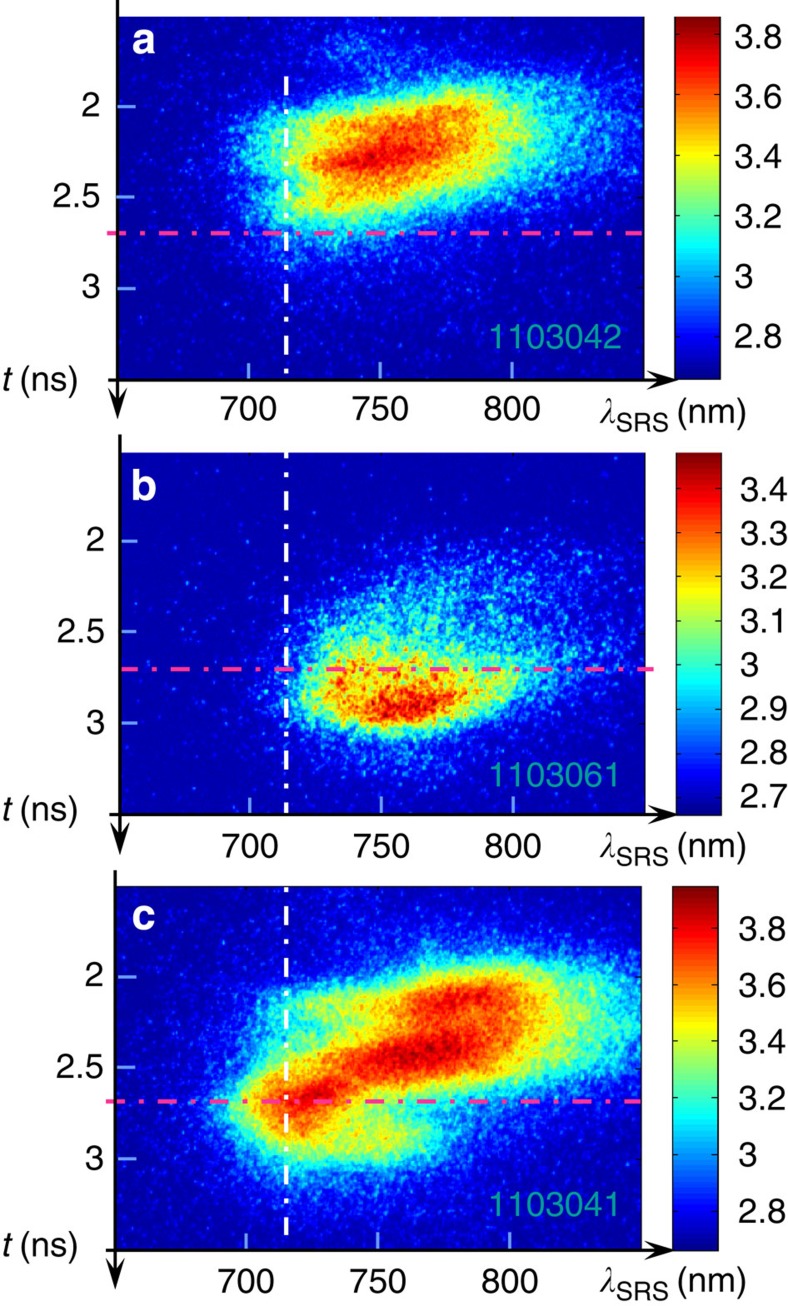
Time-resolved stimulated Raman scattering spectra. The SRS spectra are shown on a logarithmic scale for (**a**) a 6-mg cc^−1^, 300-μm foam plasma, (**b**) a 2-μm-thick CH foil and (**c**) the combination of the two. The SRS spectrum measured with the compound target (**c**) where SRS in the foam is seeded by the SRS light backscattered by the foil extends further in the short wavelengths range compared with the two other spectra (**a**,**b**). A new component is observed around *t*=2.7 ns and *λ*_SRS_=720 nm. Dot-dashed lines have been drawn on the three spectra to indicate these particular time and wavelength.

**Figure 3:**
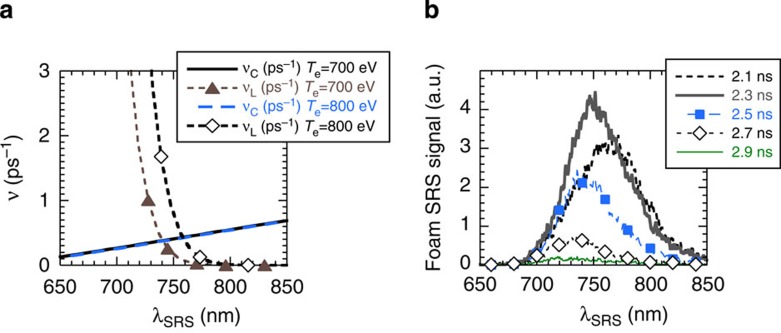
Landau cutoff in the SRS spectrum of the foam plasma. (**a**) The damping rates of the driven EPWs are shown as a function of the SRS scattered light wavelength. Collisional (*ν*_c_) and Landau (*ν*_L_) damping rates are calculated for a Maxwellian distribution function for electron temperatures of 0.7 and 0.8 keV. In the transition region where collisional and Landau damping are almost equal, at 750 nm, we calculate *kλ*_De_=0.23 for the driven EPW. (**b**) Spectrum of the SRS signal measured for the 6-mg cc^−1^, 300-μm foam-alone plasma for different times during the interaction.

As an initial step, we first characterize the SRS backscattered from a 6 mg cc^−1^ foam plasma when it starts from thermal EPW levels. The time-resolved SRS spectrum measured for a preformed foam plasma is shown in Fig. 2a. The maximum electronic density is close to 0.45 *n*_c_ when the foam is fully ionized at the end of the plasma-producing beam. The plasma then expands and the maximum density decreases. Monitoring of the half harmonic emission characteristic of two-plasmon decay instability confirms that quarter critical density (*n*_c_/4) is still present in the plasma when the interaction beam starts. SRS starts after *t*=1.8 ns, when the maximum density is already below *n*_c_/4. The maximum scattered light wavelength, which is directly connected to the maximum plasma density in which SRS develops, decreases as a function of time starting from 800 nm (or *n*_e_/*n*_c_=0.11 for 0.8 keV) at *t*=1.8 ns. In the period *t*=2.3 ns to *t*=2.6 ns, the maximum plasma density in which SRS develops decreases from 0.11 *n*_c_ at *t*=2.3 ns to 0.08 *n*_c_ at *t*=2.6 ns. A short wavelength cutoff is observed in the SRS spectrum below 720 nm in the range where the Landau damping rate, shown in Fig. 3a, sharply increases^42^,^43^,^44^. The spectrum is shown for different times during the interaction in Fig. 3b. This illustrates how the SRS growth of the foam plasma shifts as a function of time towards shorter wavelengths following its hydrodynamic expansion. The amplitude of the SRS signal also decreases simultaneously. Between its maximum at *t*=2.35 ns and *t*=2.7 ns, it drops by a factor 8 due to the density decrease that moves the amplification in a region of lower SRS gain. The temporal evolution of the transmission of the interaction beam through the foam plasma is measured in the same conditions. It starts at the beginning of the pulse, thereby validating that the foam is fully ionized at the end of the plasma heating beam. It displays an almost constant transmitted power for the full duration of the interaction beam with an average transmission of 35%. No break up of the transmitted beam is observed in the two-dimensional far-field images recorded with the charge-coupled device camera.

### Experimental results in the foil alone target

**Figure 4:**
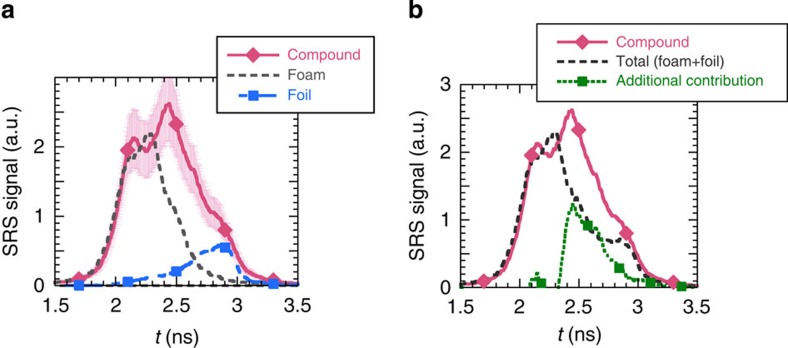
Stimulated Raman scattering signals as a function of time. (**a**) SRS signal for the three types of targets; the error bars shown for the compound target measurement illustrate the shot to shot variation; the maximum SRS reflectivity measured in the compound target is 11%. (**b**) SRS signal measured in the compound target compared with the sum of the foam-alone and foil-alone signals, and exceeding contribution observed with the compound target.

Following these observations, the interaction with the foil is studied with shots performed with the interaction beam alone fired at a reduced energy level to reproduce the laser intensity measured in the transmission of the foam alone. This is used to characterize the Raman light that comes back into the foam plasma of the compound target. The corresponding time-resolved spectrum is shown in Fig. 2b. It covers a broad spectral range extending from 720 to 830 nm. The SRS level in the foil-alone shots increases as a function of time following the foil–plasma expansion that provides longer density scalelengths more favourable to SRS growth. The electromagnetic seed level increases as a function of time on a hydrodynamics timescale. This will be used to study the amplifier response for increasing initial EPW amplitude, as it follows the slow increase of the seed intensity. The time history of the SRS reflectivities measured in single targets is summarized in Fig. 4a. In the time interval *t*=2.4–2.8 ns, SRS from the foam decreases and SRS from the foil increases.

### Experimental results in the compound target

Shots are then performed with targets where the foam is followed by the foil. The distance between the two components of the target is chosen to be 1.2 mm to eliminate hydrodynamic coupling between the two plasmas. The temporal evolution of the SRS signals measured in the compound targets is shown in Fig. 4a in comparison with the foil and foam-alone signals. Early in the pulse, the foil contribution is negligible and the SRS reflectivity in the compound target follows the one measured for the foam alone. At the end of the pulse, the foam contribution is very low and we recover the foil-alone contribution. At intermediate times (*t*=2.4 ns to *t*=2.8 ns), the compound target signal significantly exceeds the sum of the foam and foil signals as can be seen in Fig. 4b.

**Figure 5:**
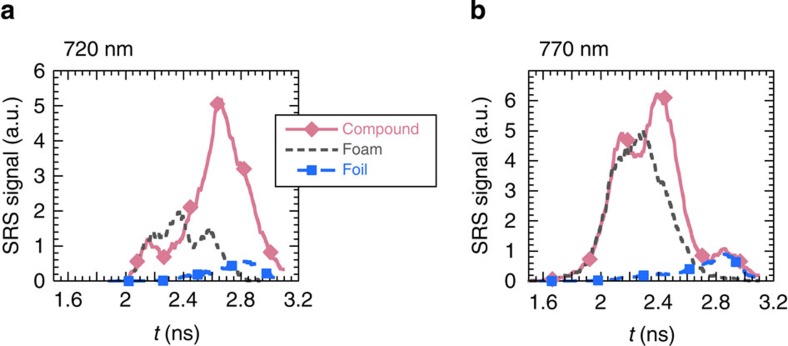
Time evolution of the SRS signals at 720 and 770 nm. The SRS signals are shown for the three types of targets in the regions dominated by either (**a**) Landau damping in the range 720±10 nm or (**b**) collisional damping in the range 770±10 nm.

Additional essential insight comes from the time-resolved SRS spectrum, in Fig. 2c, measured with the compound target: a new contribution at short wavelengths clearly appears in the time-resolved spectrum. This new component occurs at times *t*≥2.4 ns and extends from 700 to 740 nm, a wavelength range where Landau damping of the EPW dominates over collisional damping (Fig. 3a). This is also a wavelength region where the SRS from either the foam or the foil alone is very weak. The additional contribution previously evidenced in the spectrally integrated data mainly results from this new spectral component. This is clear when comparing the time evolution of the SRS signals measured at 720 and 770 nm for the three types of targets in Fig. 5a,b.

Additional shots, performed with 3 mg cc^−1^, 300-μm-long foams, lead to the same conclusions than for the 6 mg cc^−1^ foams. In the SRS signal of the 3 mg cc^−1^ foam-alone plasma, a cutoff is observed at 720 nm due to Landau damping. A new spectral component extending from 700 to 750 nm clearly appears in the spectrum of the compound target when the amplitude of the 2 μm CH SRS backscattering signal has reached a sufficient level.

### Results analysis

**Figure 6:**
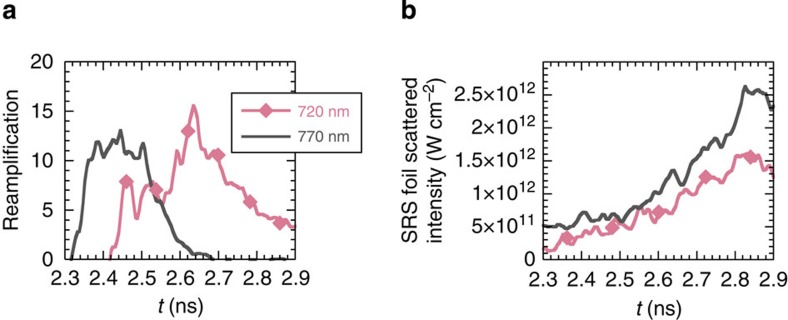
Transition to the nonlinear regime and threshold. (**a**) Reamplification as defined in the main text calculated over a wavelength range of 20 nm around 770 and 720 nm as a function of time. The transition in the region dominated by Landau damping (720 nm) is observed at *t*=2.58 ns, (**b**) time history of the foil SRS reflected intensity measured at 720 and 770 nm. At *t*=2.58 ns, the wave scattered at 720 nm reaches an intensity of *I*_SRS,foil_≈5 × 10^11^ W cm^−2^.

The additional contribution observed in the 6 mg cc^−1^ foam in the period *t*=2.4 ns to *t*=2.8 ns in the shots with the compound target is interpreted as the reamplification of the foil signal as it propagates through the foam plasma, with a reamplification factor defined as (*SRS*_compound_−*SRS*_foil_−*SRS*_foam_)/*SRS*_foil_. It is extracted from the data for different wavelength ranges and plotted as a function of time in Fig. 6a. At 770 nm, the reamplification starts at *t*=2.3 ns, stabilizes at (11±1) for 150 ps and then decreases. At 720 nm, the reamplification starts at *t*=2.4 ns and first stabilizes at a slightly lower value of 6. For *t*>2.58 ns, we observe a sharp increase in the reamplification at 720 nm that rapidly exceeds the one observed earlier at any wavelength: in <200 ps, the reamplification at 720 nm rises from 6 to 16. The reamplification observed for *t*<2.58 ns is in agreement with convective amplification of the seed signal in the foam plasma, with an associated small gain (~2–3) that decreases for shorter scattered light wavelengths due to the increased EPW damping rate in this wavelength range (see Fig. 3a). Such convective amplification of the small foil seed signal in the foam plasma results in the continuation of the foam SRS activity for longer time in the compound target. The change in reamplification observed at *t*~2.6 ns at 720 nm occurs when the linear amplification of unseeded SRS from the foam plasma is decreasing for all wavelengths, including short ones (see Fig. 3b). The sudden change in the nonlinear reamplification of the large seed would not be expected if the growth rate remained the linear one that evolved in time due to hydrodynamic evolution of the foam plasma. Further, the nonlinear change is seen to occur only in the wavelength range where Landau damping dominates over collisional damping, which is a signature of the nonlinear Langmuir wave response resulting from trapping effects. On the timescale of this experiment, ion wave nonlinearities could also affect the EPW evolution. The effect of coupling with the ion acoustic wave dynamics would be to saturate the amplitude of the Langmuir waves as they loose part of their energy in the secondary Langmuir decay instability (LDI). LDI is the decay of an EPW into a counterpropagating EPW and an ion acoustic wave^45^,^46^,^47^. Past Thomson scattering measurements performed in the transition region between fluid and kinetic nonlinearities that we explore here have shown significant LDI only in the fluid regime^48^. Thereby, in our experiment the only possible effect of LDI of the driven EPW could be to limit the SRS amplification factor measured at 770 nm.

Our experiment has been specially designed to investigate the nonlinear response of driven EPWs with a setup allowing the direct measurement of the threshold for trapping effects to influence the SRS amplification. Measurements have been performed below and above the threshold for nonlinear effects in the kinetic regime and compared with similar measurements performed in the fluid regime. This way of proceeding allows us to clearly identify the nonlinear behaviour of EPWs in the kinetic regime as responsible for the change in the SRS behaviour at short wavelength. Our experiment demonstrates an enhanced SRS amplification compared with convective amplification in this regime in relevant experimental situations with controlled conditions. The associated effects can be observed as soon as the foil contribution in the corresponding wavelength range has reached a threshold level of ~5 × 10^11^ W cm^−2^ as can be seen in Fig. 6b. This threshold is set by the competition between trapping-induced modifications of the electron distribution function and relaxation mechanisms that tend to restore a Maxwellian distribution such as collisions and escape from the speckles.

## Discussion

These observations can be discussed in the context of ICF experiments. The target geometry used for the present experiment reproduces the geometry of multiple plasmas inside a hohlraum along a laser beam path: the underdense foam mimics the hohlraum gas and the CH foil, the ablator or hohlraum wall. The reflected foil intensity above which nonlinear effects in the Langmuir wave behaviour are observed corresponds to a small SRS reflectivity of 0.1% at 720 nm. Our results indicate that a very small level of reflectivity produced deep inside the cavity may significantly modify the conditions for the amplification of the backscattered SRS light on its path towards the laser entrance hole. The plasma combination results in a significant SRS contribution in the short-wavelength range. As the origin of this effect is in the kinetic nonlinearities of the Langmuir wave, this result would not be expected from standard linear gain calculations. In the geometry of ICF hohlraum targets, a similar situation may occur when light is backscattered from the wall or ablator plasma and then propagates through the gas plasma before exiting the cavity^37^,^38^,^39^,^49^. Experimental SRS spectra reported for National Ignition Facility experiments at the megajoule level include contributions at short scattered light wavelengths that sometimes exceed the contributions at longer wavelengths. Although the global shape of the spectra is rather well reproduced by linear gain calculations^39^, a striking feature is that the ratio between the short-wavelength early time and long-wavelength late time contributions is clearly underestimated: the level of SRS gain at short wavelengths (*λ*_SRS_<540 nm) is not reproduced by the simulations^39^ despite improvements in the radiative hydrodynamics simulations that generate the plasma conditions. Linear reamplification of the light backscattered in the target interior by multiple crossing beams in the region of the laser entrance hole has been used to explain the enhanced contribution at short scattered light wavelengths^37^,^38^. Our experiment, performed in a simpler geometry, clearly demonstrates an additional effect that enhances the shortest wavelength range contribution. Our interpretation involves nonlinearities in the plasma wave response so that the final SRS signal not only results from the reamplification of a seed but also from an increased SRS gain modified by kinetics effects that should also be included in the simulations to reproduce the experimental results.

The threshold intensity of the counterpropagating wave above which this effect should be considered in the simulations is deduced solely from the experiment. It can be compared with theoretical estimates developed by Strozzi *et al*.^50^ Their model considers three-dimensional detrapping effects as well as collisions. It compares the time needed before trapped electrons can significantly affect the electron distribution function with the detrapping time. Depending on the conditions, this is fixed by the collisional relaxation of the distribution function or by the time taken by the electrons to escape the interaction region, whose dimensions are fixed by the focusing optics. The amplitude of the EPW produced by the ponderomotive force of the two counterpropagating electromagnetic waves locally grows like *δn/n*=1/4 × *v*_1_ × *v*_2_ × *k*_EPW_^2^/*ω*_p_*t* (refs 51, 52) where *v*_1_ and *v*_2_ are the maximum oscillating velocities in the electric fields of the incident laser and of the light backscattered by the foil plasma, *k*_EPW_ is the wave–vector module of the driven EPW and *ω*_p_ is the plasma frequency. For our conditions, using the average incident laser intensity and the average threshold intensity of *I*_SRS,foil_≈5 × 10^11^ W cm^−2^, we calculate from the expression of *δn/n* that the time needed for trapping to distort the electron distribution function significantly is *τ*_trap_~0.2 ps. At this time, the EPW has reached an amplitude of *δn/n*=7 × 10^−3^. In the meantime, the trapped electrons would escape the speckle on longer characteristic times ~0.3 ps. This indicates that our experimental threshold corresponds to the limit where trapping effects start influencing the EPW evolution according to ref. 50. As *τ*_trap_ decreases slowly for increasing intensity, considering speckles with higher intensity in the incident laser or backscattered light will not significantly affect this conclusion: below threshold for the more intense speckles, no evidence of trapping is seen in the experiment, and as soon as the most intense speckles exceed the threshold the lowest intensity ones will also exceed it for a seed intensity just 1.5 times higher. As a consequence, the intensity variations in the random phase plate (RPP) focal spot do not affect our conclusion. Our experimental results thus validate the theoretical estimates presented in ref. 50. They also demonstrate that close to threshold, the reduction in Landau damping dominates over frequency detuning and other trapped particle instabilities so that the SRS amplification factor increases compared with the convective one. The key parameters that settle the impact of kinetic nonlinearities of the driven Langmuir waves on the SRS amplification factor in the regime initially dominated by Landau damping are the electron temperature and the speckle size that both determine the characteristic time for side loss, which must be compared with *τ*_trap_. For the experiments on the National Ignition Facility, where large levels of stimulated Raman scattering were measured, we calculate that detrapping through side loss is effective in 0.15 ps, which is a situation very close to our experiment.

Our experiment is performed in the nanosecond regime but its counterpropagating geometry is similar to the one used for the amplification of short pulses in BRA schemes. There, the two electromagnetic waves correspond to the long pump and short seed pulses. Our experiment demonstrates that low seed levels are sufficient to trigger trapping-induced nonlinearities. The nonlinear effects observed in our experiment develop on times that shorten as the intensity of the seed increases^33^: as soon as the seed intensity exceeds 10^16^ W cm^−2^, we calculate *τ*_trap_<50 fs. As a result, initial strong linear Landau damping rates could be reduced by trapping effects over the duration of the seed pulse, allowing the BRA scheme to operate in this regime of strong linear Landau damping^53^. However, our observations of increasing amplification with seed intensity were not obtained in experiments^37^,^38^,^54^ when the seed intensity was scanned in the range we explored. Although trapping effects develop in ~0.3 ps, the low electron temperature of the plasma and the laser parameters result in longer times (~1.7 ps) for trapped particle to escape the speckle. Experiments with shorter-wavelength laser in higher-temperature plasmas may reach a regime similar to ours for the amplification of picosecond pulses. In these experiments, the shifted seed pulse may be directly generated in the targets similar to that in our experimental setup. Although BRA uses SRS in plasmas for the amplification of short pulses, the initial seed beam energy remains limited by the use of Raman crystals or gas cells. Our experiment suggests that the seed pulse may be produced by direct interaction of the unshifted short-pulse laser in a plasma (see Methods for details).

Our experimental demonstration that trapping nonlinearities develop for small levels of ponderomotive drive may explain some of the past experiments that reported increased stimulated Raman scattering reflectivities compared with those expected from linear gain calculations^46^. Our observations are of prime interest to the ICF community as they contribute to explain the large level of stimulated Raman backscattering measured in experiments at the megajoule level. The experiment was designed to provide an experimental answer in a transition region where trapping nonlinearities are close to threshold, and where collisional and Landau damping rates are of the same order. Although relevant to experiments performed in the context of BRA and ICF (see the Methods section), these conditions are among the most complicated to describe with numerical simulations. As a consequence, our measurement of the nonlinear response of a driven plasma wave in this regime is likely to guide future theoretical and numerical studies.

## Methods

### Laser beams and diagnostics configuration

The experiment is performed with the two kJ beams of the LULI 2000 laser facility at Ecole Polytechnique, both converted to the second harmonic (*λ*_laser_=526.5 nm) with a 50% conversion efficiency. The pulse has a duration of 1.5 ns for each beam with a time delay between the two beams of 1.5 ns. The first beam is used as a heating beam to ionize the foam target. It is smoothed with a RPP with 4-mm square elements and focused by a 1.6-m, f/8 focal lens with a resulting average intensity on target equal to 3 × 10^14^ W cm^−2^ in the 250-μm focal spot. The second beam then interacts with the preformed foam plasma followed in some cases by the foil. It is smoothed with an RPP with 5 mm square elements and focused by a 1.6-m, f/8 focal lens with a resulting average intensity on target of 5 × 10^14^ W cm^−2^ in the 200-μm focal spot. Two optical stations are set up on this interaction beam to analyse both the transmitted and reflected lights. The transmission is collected by an f/4 lens and imaged onto a charge-coupled device, a streak camera and a fast photodiode. The backscattered SRS light is collected from the reflection on an uncoated plate inserted on the incident interaction beam. It is analysed by the combination of a spectrometer and a streak camera in the Raman (*λ*∈[*λ*_laser_; 2*λ*_laser_ ]) wavelength range with a spectral resolution of 20 nm and a temporal resolution of 200 ps. The time-integrated Raman backscattered energy is measured with a fast photodiode. Shots were performed with either the foam or the foil alone, and with the contribution of the two. The comparison of the Raman scattering levels for three conditions is used to measure the reamplification factor, as defined in the text, of the foil signal in the foam plasma.

### Generation of the shifted seeding electromagnetic wave in a plasma

**Figure 7:**
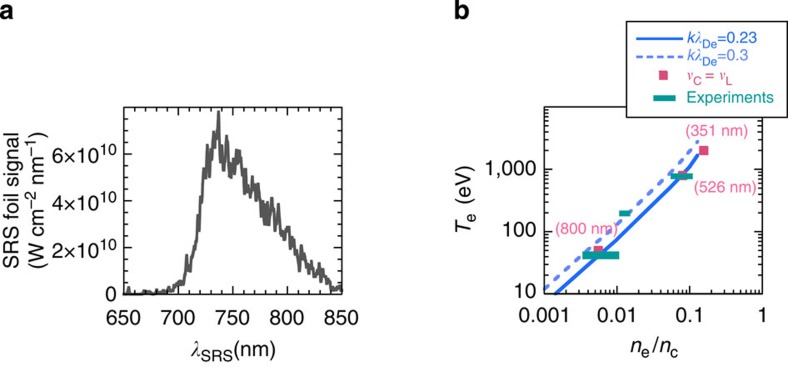
Characteristics of the electromagnetic seed and amplifier plasma. (**a**) The spectral intensity of the foil SRS signal is shown at *t*=2.6 ns. With integration over a 500-μm spot size and a 5-ps duration, it peaks at 0.59 mJ nm^−1^, which represents better performances than reached in Raman gas cells^54^, (**b**) The *kλ*_De_=0.23 and *kλ*_De_=0.3 curves are plotted in the (*n*_e_, *T*_e_) plane. The parameters values for which *ν*_c_ equals *ν*_L_ in a Maxwellian plasma are also shown for *T*_e_=2 keV for 351 nm laser wavelength, for *T*_e_=0.8 keV at 526 nm and for *T*_e_=50 eV at 800 nm. The experiments shown in this graph are those of ref. 35 close to 50 eV, of ref. 54 close to 200 eV and the one described in this paper close to 0.8 keV.

The counterpropagating electromagnetic seed is produced by direct interaction of the interaction beam with a 2-μm-thick CH foil. The latter has been chosen because for the interaction beam parameters (*λ*_laser_=526.5 nm and *I*_0_=5 × 10^14^ W cm^−2^), it is fully ablated in ~0.5 ns. SRS of the interaction beam in the expanding foil plasma is an efficient way to generate the shifted electromagnetic wave. The spectral intensity of the SRS signal from the foil plasma is shown in Fig. 7a. The laser intensity is converted with a maximum conversion efficiency of 5% in a wave shifted to 760 nm covering a narrow bandwidth of <100 nm. This represents better performances than Raman gas cells, which produce a shifted energy distributed over a wider spectrum (~300 nm). If calculated as a rate of conversion per unit of wavelength, Raman scattering in a plasma is as efficient as Raman crystals, except that this new technique can handle higher energy pulses: the shifted signal is produced in a diameter equivalent to the incident laser beam whose intensity can be up to a few 10^15^ W cm^−2^ without significant broadening of the SRS spectrum. For completeness, the performances of this new scheme have been tested in the picosecond regime: it was found that equivalent performances can be reached with a 5-ps pulse interacting with a foil preformed plasma.

### The foam plasma amplifier

The main foams chosen for this study were 6 mg cc^−1^, 300 μm long. The ionization of these foams was diagnosed through time-resolved measurements of the transmission and through transverse imaging of the ionization front. Both measurements are consistent and give an average velocity of 2 × 10^7^ cm s^−1^ validating that the ionization is supersonic for these foam parameters. When fully ionized at *t*=1.5 ns, the 6 mg cc^−1^ density results in a 0.45 *n*_c_ electronic density. SRS of the interaction beam provides the amplification of this plasma when starting from noise and indicates that the foam plasma response extends into the range of interest (720–770 nm) for times >2.4 ns when the foil SRS signal has reached a sufficient level. With this choice of foam plasma amplifier, the transition region between the two regimes, respectively, dominated by collisional and Landau damping could be investigated. In our experiment, the transition region corresponds to *k*λ_De_~0.23. Owing to the sharp increase in the Landau damping rate with *k*λ_De_ in this region, it equals the collisional damping rate close to *k*λ_De_~0.23–0.3 over a wide range of density and temperature. This is shown in Fig. 7b which illustrates that the transition region described in ref. 55 and studied in this experiment is a general feature of numerous BRA and ICF experiments.

## Author contributions

The experiment was performed by S.D., V.Y., C.G., G.L., C.L., O.R. and T.R. A.O. and N.B. prepared the foam targets. V.Y., C.G. and G.L. designed and operated the transmission and backscattering diagnostics. O.R. and T.R. designed and operated the X-ray diagnostics used for plasma characterization. S.D. and V.Y. carried out the data analysis. S.D. interpreted the results. The interpretation was discussed with C.L. and P.-E.M.-L. S.D. and C.L. wrote the paper.

## Additional information

**How to cite this article:** Depierreux, S. *et al*. Laser light triggers increased Raman amplification in the regime of nonlinear Landau damping. *Nat. Commun.* 5:4158 doi: 10.1038/ncomms5158 (2014).
